# Enrichment of patients with concomitant limbic‐predominant age‐related TDP‐43 encephalopathy (LATE) on the Alzheimer's disease continuum using hippocampal volume

**DOI:** 10.1002/alz.70970

**Published:** 2025-12-13

**Authors:** Nidhi S. Mundada, Xueying Lyu, Christopher A. Brown, Niyousha Sadeghpour, Emily McGrew, Long Xie, Philip A. Cook, James Gee, Paul A. Yushkevich, Sandhitsu R. Das, David A. Wolk, for the Alzheimer's Disease Neuroimaging Initiative

**Affiliations:** ^1^ Department of Bioengineering University of Pennsylvania Philadelphia Pennsylvania USA; ^2^ Department of Neurology Perelman School of Medicine at the University of Pennsylvania Philadelphia Pennsylvania USA; ^3^ Department of Radiology Perelman School of Medicine at the University of Pennsylvania Philadelphia Pennsylvania USA

**Keywords:** aging, Alzheimer's disease, co‐pathology, LATE, structural MRI, TDP‐43

## Abstract

**INTRODUCTION:**

Clinical overlap between Alzheimer's disease (AD) and limbic‐predominant age‐related transactive response DNA binding protein 43 (TDP‐43) encephalopathy (LATE), combined with the absence of validated in vivo biomarkers, complicates identification of mixed AD/LATE pathology. We labeled individuals along the AD continuum with suspected concomitant LATE using the lower quartile hippocampal volume (HV) cut‐off and examined associated atrophy and cognitive profiles.

**METHODS:**

We studied cognitively impaired (CI) Alzheimer's Disease Neuroimaging Initiative (ADNI) participants with T1‐magnetic resonance imaging (MRI) and amyloid‐ and tau‐positron emission tomography (PET). Participants were classified into suspected (s) AD+sLATE−, AD−sLATE+, or AD+sLATE+ based on amyloid status and HV quartiles. Medial temporal lobe (MTL) and whole‐brain atrophy patterns and cognitive profiles were compared cross‐sectionally and longitudinally. Classification was validated in an autopsy cohort.

**RESULTS:**

AD+sLATE+ showed greater anterior hippocampal (AH) and amygdala atrophy than AD+sLATE−. AD−sLATE+ and AD+sLATE+ showed greater anterior MTL atrophy and worse memory and language performance. AD+sLATE+ also exhibited faster cognitive decline.

**DISCUSSION:**

A simple HV quartile cut‐off may help identify mixed AD/LATE pathology and support clinical trial enrichment.

**Highlights:**

- Quartiles of HVs stratify CI individuals.
- Approach distinguishes suspected AD+sLATE–, AD–sLATE+, and AD+sLATE+ subgroups.
- The method is simple, scalable, and requires no complex modeling or biomarker panels.
- It enables practical identification of mixed pathology in clinical settings.
- It supports trial enrichment by excluding or targeting mixed pathology cases.

## 1 BACKGROUND

Alzheimer's disease (AD) is the leading cause of dementia, characterized by progressive cognitive decline driven by the accumulation of amyloid beta (Aβ) plaques and tau neurofibrillary tangles.^1^ While Aβ deposition is an early hallmark of AD pathology, tau tangles are more strongly associated with neuronal loss and clinical progression, positioning tau as the primary driver of neurodegeneration in AD.^2^, ^3^, ^4^, ^5^ However, many individuals with AD harbor additional age‐related changes and neuropathologies that contribute to cognitive decline and neurodegeneration.^6^, ^7^, ^8^

One such co‐pathology is limbic‐predominant age‐related transactive response DNA binding protein 43 (TDP‐43) encephalopathy (LATE), increasingly recognized as a major contributor to memory impairment in older adults.^9^ LATE neuropathic change (LATE‐NC) primarily involves limbic structures such as the hippocampus and amygdala and is characterized by the pathological aggregation of TDP‐43.^10^, ^11^, ^12^ While its clinical presentation often resembles that of AD, LATE‐NC follows a distinct progression trajectory and frequently coexists with AD pathology, complicating differential diagnosis. The high prevalence of LATE among individuals with AD – estimated at up to 50% of advanced AD (Braak stage V–VI) in community‐based autopsy cohorts^13^ – limits attribution of atrophy and clinical symptoms solely to AD pathology, given that standard biomarkers capture amyloid and tau but not TDP‐43 pathology. Furthermore, individuals with both AD and LATE pathologies experience faster cognitive decline and greater neurodegeneration than those with AD alone.^14^, ^15^, ^16^ This raises additional questions about whether LATE‐NC co‐pathology modulates response to AD‐targeted treatments, such as anti‐amyloid immunotherapy.^17^ These uncertainties underscore the need for better stratification methods to identify individuals with likely LATE co‐pathology so as to improve diagnostic precision and guide treatment and trial enrollment.

Identifying LATE during life remains challenging due to the absence of clinically available positron emission tomography (PET) or biofluid biomarkers for TDP‐43 pathology, though promising candidates are under development.^18^, ^19^ Generally, investigators have examined indirect markers of LATE‐NC – including both structural^20^, ^21^ and metabolic^22^, ^23^, ^24^ neuroimaging – to differentiate LATE from AD. While hippocampal atrophy is a hallmark of both AD neuropathological change (ADNC) and LATE‐NC, Yu et al.^25^ demonstrated that autopsy‐confirmed cases with ADNC+LATE‐NC or LATE‐NC alone exhibited significantly smaller *post mortem* hippocampal volumes (HVs) than those with ADNC alone. Notably, among individuals with ADNC, those in the lower range (∼lowest quartile) of HV appeared disproportionately enriched for concomitant LATE‐NC, suggesting that TDP‐43 contributes to atrophy variability within AD. These findings indicate that TDP‐43 pathology contributes to neurodegeneration beyond what is explained by amyloid and tau burden and may explain observed heterogeneity within the AD continuum. Indeed, a recent consensus group proposed clinical criteria for diagnosing possible or probable LATE, emphasizing not only memory impairment but also structural magnetic resonance imaging (MRI) evidence of substantial hippocampal atrophy.^26^

While prior studies proposed MRI‐based predictors of LATE‐NC,^20^, ^21^, ^27^, ^28^ these often involve complex multivariate models that may be difficult to implement in clinical settings. Motivated by *post mortem* imaging findings,^25^ we adopted HV quartiles as a heuristic, in vivo framework to stratify individuals along the AD continuum by suspected LATE co‐pathology. Unlike multivariate or PET‐dependent approaches, this simple method relies on a single, widely available structural MRI measure. Our goal was to establish this quartile‐based classification in in vivo imaging, evaluate biomarker and clinical correlates, and validate the enrichment of suspected LATE pathology in an autopsy‐confirmed cohort. We defined the lowest quartile as atrophy beyond what is “typically” observed in AD, expected partly due to underlying LATE co‐pathology. Furthermore, we hypothesized that these individuals would exhibit distinct structural MRI patterns linked to LATE‐NC (e.g., greater anterior‐to‐posterior gradient of medial temporal lobe [MTL] atrophy)^21^, ^29^, ^30^ and clinical features such as accelerated cognitive decline.^14^ To assess the pathological validity of this approach, we further evaluated it in an independent autopsy cohort. By refining identification of individuals along the AD continuum – particularly those with concomitant LATE co‐pathology – this study aims to advance our understanding of neurodegenerative heterogeneity. This approach has the potential to enhance cohort selection in research, refine biomarker studies, and contribute to more personalized strategies for diagnosing and treating mixed‐etiology dementia.

## 2 METHODS

### 2.1 Participants

#### 2.1.1 Main cohort

Participants were retrospectively selected from the Alzheimer's Disease Neuroimaging Initiative (ADNI) study (http://adni.loni.usc.edu). We applied a HV cut‐off to cognitively impaired (CI) individuals who met the following criteria: (1) clinical diagnosis of mild cognitive impairment (MCI) or dementia and (2) amyloid‐PET, tau‐PET, and 3T T1‐weighted MRI scan within 365 days of each other. Amyloid positivity was determined based on amyloid‐PET: 232 individuals were classified as amyloid‐positive (Aβ+), and 165 individuals were classified as amyloid‐negative (Aβ–). Enrichment criteria were applied to the total sample (refer to Section 2.3 on details of stratification and Figure S1 for cohort selection flowchart). In addition, we included Aβ– cognitively unimpaired (CU) individuals who were matched to the CI group on age, sex, and education and had complete imaging data to serve as a reference group.

**RESEARCH IN CONTEXT**

1. **Systematic review**: We conducted a literature review using PubMed, and recent conference proceedings focused on hippocampal atrophy in AD, LATE, and mixed pathologies. Prior studies reported on the overlap of hippocampal involvement across these conditions, but few have proposed simple, scalable methods to stratify patients in a clinically applicable manner.
2. **Interpretation**: Our findings show that a quartile‐based cut‐off on HV can effectively stratify CI individuals into suspected AD+sLATE–, AD+sLATE+, and AD−sLATE+ groups. This simple classification framework offers a clinically scalable strategy for identifying mixed pathology and can aid in enriching clinical trial cohorts by either excluding or specifically targeting individuals with suspected co‐existing LATE pathology.
3. **Future directions**: Validation of this approach in diverse cohorts is needed. Future studies should evaluate its utility in predicting therapeutic response and in refining inclusion criteria for disease‐modifying trials targeting amyloid, tau, or TDP‐43.

#### 2.1.2 Autopsy cohort

ADNI participants with neuropathologic assessments and T1‐weighted MRI, either 1.5 or 3T, were included as an independent validation cohort. Using neuropathologic assessments, we characterized individuals with a definitive diagnosis using their ADNC assessments and presence of TDP‐43 in their amygdala with or without spread to hippocampus/entorhinal cortex (ERC) or beyond (TDP‐43 stage ≥ 1 as defined by Nelson et al).^10^ Individuals with intermediate/high ADNC and TDP‐43 stage < 1 were classified as AD+LATE− (*n* = 44) and those with intermediate/high ADNC and TDP‐43 stage ≥ 1 as AD+LATE+ (*n* = 35). Of the 79 participants, 69 were CI at the time of their baseline scan, while 10 were CU, eight of whom converted to CI prior to death. Because the aim of this study was to evaluate whether HV quartiles enrich for suspected LATE within the context of AD pathology, we restricted analyses to AD+LATE– and AD+LATE+ groups. While other co‐pathologies may also contribute to hippocampal atrophy, our focus on these groups allowed us to isolate the contribution of TDP‐43 within the AD continuum.

### 2.2 Image acquisition and processing

#### 2.2.1 Image acquisition

Individuals in the main analysis had 3T T1‐weighted MRI scans of resolution 1.0 × 1.0 × 1.2 mm^3^ or 1.0 × 1.0 × 1.0 mm^3^, while those in the autopsy sample had either 1.5T T1‐weighted MRI (*n* = 39) of resolution 1.25 × 1.25 × 1.2 mm^3^ or a 3T T1‐weighted MRI (*n* = 40) of resolution 1.0 × 1.0 × 1.2 mm^3^ or 1.0 × 1.0 × 1.0 mm^3^.

Amyloid‐PET imaging was acquired using either ^18^F‐Florbetapir (50–70 min after injection) or ^18^F‐Florbetaben (90–110 min after injection). Tau‐PET imaging was performed using six 5‐min frames acquired between 75 and 105 min after injection of ^18^F‐Flortaucipir. Preprocessed tau‐PET images with a uniform 6 mm full‐width‐at‐half‐maximum (FWHM) resolution were obtained from the ADNI archive (“Coreg, Avg, Std Img and Vox Size, Uniform Resolution”).

#### 2.2.2 T1‐MRI processing

The automatic segmentation of hippocampal subfields‐T1 (ASHS‐T1, https://sites.google.com/view/ashs‐dox/) pipeline was used to automatically segment the MTL subregions.^31^ Segmentations were performed on each participant's T1‐MRI scan closest in time to their tau‐PET scan (used for cross‐sectional analyses), as well as all available T1‐MRIs within ±5 years from the scan used for cross‐sectional analyses. The segmented subregions included the anterior and posterior hippocampus (AH/PH), amygdala, ERC, Brodmann areas 35 and 36 (BA35 and BA36), and the parahippocampal cortex (PHC). ASHS‐T1 is specifically designed to address confounds like inclusion of dura mater, which can limit traditional whole‐brain segmentation methods.^32^ For each subregion, a summary region of interest (ROI) metric was extracted: Volumetric measurements were computed for AH, PH, and amygdala, while median cortical thickness was derived for the MTL cortical regions (ERC, BA35, BA36, and PHC) using the “cortical reconstruction for ASHS (CRASHS)” surface‐based pipeline applied to the ASHS‐T1 output. Details of the CRASHS pipeline have been outlined here.^33^ Briefly, CRASHS applies surface‐based cortical modeling and diffeomorphic registration to ASHS‐T1 segmentations, enabling the extraction of median cortical thickness for MTL cortical regions, as well as pointwise thickness maps, with consistent anatomical correspondence across participants enabling vertex‐wise statistical analyses of regional disease effects. Of the 2554 thickness maps from the main analysis, left and right separate, from 386 participants, including cross‐sectional and longitudinal time points, 0.7% failed processing. Intracranial volume (ICV) was estimated from each participant's structural MRI using in‐house segmentation software built on the ASHS framework.

Additionally, T1‐weighted MRI scans were bias‐corrected and skull‐stripped using Advanced Normalization Tools (ANTs), followed by cerebellar, cortical, and subcortical parcellation via multi‐atlas Joint Label Fusion with the BrainColor atlas, which defines 102 ROIs.^34^, ^35^ Whole‐brain thickness maps were generated using the DiReCT cortical thickness estimation method.^36^ For longitudinal analyses, T1‐weighted scans were processed using a longitudinal ANT‐based pipeline that incorporates symmetric diffeomorphic registration to generate within‐subject templates and ensures consistent cortical thickness estimation across time points, as validated in prior work.^37^

For longitudinal analysis, we included scans ±5 years from the time point used in the cross‐sectional analyses. Among 386 participants, 304 had longitudinal MRI scans, with an average of 3.9 ± 2.1 scans per participant over a follow‐up span of 0.5 to 9.8 years.

#### 2.2.3 PET processing

Rigid‐body registration of tau‐PET to T1‐weighted MRI was performed using ANTs,^38^ and all registrations were visually inspected for quality assurance. Standardized uptake value ratio (SUVR) maps were computed using an inferior cerebellar reference region, and mean SUVR values were extracted from all cortical and subcortical ROIs following partial volume correction.

To quantify tau burden, Tau‐MaX, a combined measure of tau extent and magnitude, was computed using a Gaussian mixture modeling approach to distinguish pathologic from non‐pathologic signal from tau‐PET scans. Details on computing Tau‐MaX have been described elsewhere.^39^ Tau‐MaX values were calculated for both a global cortical region set and a temporal meta‐ROI, yielding global and temporally specific Tau‐MaX measures used in subsequent analyses.

Amyloid positivity was determined using amyloid‐PET SUVRs provided by the ADNI PET Core. Patients were classified as Aβ+ based on predetermined thresholds^40^: SUVR ≥ 1.11 for ^18^F‐Florbetapir and 1.08 for ^18^F‐Florbetaben.

### 2.3 Cohort stratification: percentile‐based grouping

HV quartiles were derived from the distribution of HVs among Aβ+ participants, reflecting our primary aim to stratify suspected LATE within the AD continuum. This choice was motivated by prior *ex vivo* work,^25^ which demonstrated that ADNC positive cases with concomitant LATE were disproportionately represented among those with the smallest HVs, suggesting that TDP‐43 pathology contributes to the lower tail of the HV distribution in AD. Because Aβ− individuals in ADNI represent a more heterogeneous group – including non‐AD pathologies and normal aging – and our available CI Aβ− sample was relatively small, we did not derive thresholds from that group. Instead, we applied the Aβ+‐derived cut‐off to Aβ− participants for exploratory comparison, using the CI Aβ− group as a reference for suspected LATE‐only phenotypes and to assess the degree of overlap with the AD+sLATE+ group.

We assessed the distribution of total HV (AH + PH) in patients with MCI or dementia due to AD. We used the total HV of the most affected hemisphere, that is, the smaller of the two HVs, rather than taking a mean of the two hemispheres, as LATE‐NC is frequently asymmetric.^10^ Age at MRI and ICV‐adjusted HV was calculated for all patients using the regression estimates from all CU individuals; sex was not included as a covariate since ICV accounted for sex differences.

Using a quartile‐based approach, we classified Aβ+ patients into suspected (s) “AD+sLATE–” and “AD+sLATE+” groups based on the distribution of adjusted HV among Aβ+ patients. Patients in the first quartile (Q1; percentile < 25) were classified as “AD+sLATE+,” and patients in the third and fourth quartiles (Q3–Q4, percentile > 50) were classified as “AD+sLATE–.” Patients in the second quartile (Q2; 25 < percentile < 50) were excluded.

We included two Aβ− reference groups, Aβ–CI and Aβ–CU. The same cut‐off from the Aβ+ patients was applied to the Aβ–CI participants to select patients with significantly low HV (Q1). Given this level of hippocampal atrophy, these patients were suspected to have LATE‐NC as the primary underlying pathology, identified as “AD−sLATE+” in this study. The Aβ−CU group served as an additional reference group.

In summary, we had four groups, two disease‐of‐interest groups defined using the quartile approach for HV, (1) AD+sLATE– (Aβ+CI, Q3–Q4), (2) AD+sLATE+ (Aβ+CI, Q1), and two reference groups, (3) AD−sLATE+ (Aβ−CI, Q1), and (4) Aβ−CU.

### 2.4 Statistical analysis

Statistical analyses were performed using R version 4.4.2 (www.r‐project.org), except for cross‐sectional pointwise MTL thickness analyses, which were carried out in Python with the meshglm tool from the CM‐Rep package (github.com/pyushkevich/cmrep) and cross‐sectional voxel‐wise whole‐brain analyses, which were carried out using FSL tools.^41^ All tests were two‐sided.

#### 2.4.1 Region‐level analyses and imaging features

We assessed group differences in ROI volumes (AH, PH, and amygdala) and derived imaging features using pairwise *t* tests. Imaging features were selected based on a priori research suggestive of the presence of LATE‐NC. Ratio of ERC/PHC thickness was computed, where lower ratios suggest more thinning in the anterior MTL. Since LATE is often asymmetric, we calculated hippocampal asymmetry index as follows: abs([L–R]/[L+R] × 200), where higher values indicate greater asymmetry between the two hemispheres. All values are positive since we take the absolute value.

#### 2.4.2 MTL pointwise analyses

For MTL pointwise group comparisons, general linear model (GLM) testing was conducted at each vertex of the cortical thickness maps. Permutation testing using the threshold‐free cluster enhancement (TFCE) approach^42^ with parameters *E* = 0.5, *H* = 2, and Δ*h* = 0.01, and 10,000 permutations were used to compute family‐wise error rate (FWER)‐corrected *p* values at each vertex. Age was used as a covariate when comparing the AD+sLATE–, AD−sLATE+, and AD+sLATE+ groups to Aβ–CU; age and temporal Tau‐MaX were used as covariates when comparing AD+sLATE– versus AD+sLATE+ groups to control for the effect of tau pathology, a surrogate for AD progression, for cross‐sectional analyses. For longitudinal analyses, we fit a linear mixed‐effects (LME)^43^ model at every vertex with random intercepts with false discovery rate (FDR) correction. Covariates included are in the following model equations:

$$\begin{array}{ccc} & & \text{CI}\;\text{versus}\;A\beta -\text{CU}:\text{Thickness}∼\text{time}+\text{group}+\text{age}\;\text{at}\;\text{baseline}\;\text{scan}\\ & & \;+\;\text{time}\times \text{group}+(1|\text{ID}) \end{array}$$

$$\begin{array}{ccc} & & \text{AD}+\text{sLATE}-\text{versus}\;\text{AD}+\text{sLATE}+:\text{Thickness}∼\text{time}\\ & & \;+\;\text{group}+\text{age}\;\text{at}\;\text{baseline}\;\text{scan}+\text{temporal}\;\text{Tau}-\text{MaX}\\ & & \;+\;\text{interval}\;\text{between}\;\text{baseline}\;\text{MRI}\;\text{and}\;\text{tau}-\text{PET}+\text{time}\\ & & \;\times \;\text{group}+(1|\text{ID}) \end{array}$$

#### 2.4.3 Whole‐brain analyses

For whole‐brain voxel‐wise cross‐sectional analyses, cortical thickness maps were analyzed using the TFCE method with parameters *E* = 0.5, *H* = 2, and Δ*h* = 0.01 and 10,000 permutations using FSL randomize. Age was used as a covariate for AD+sLATE–, AD−sLATE+, and AD+sLATE+ versus Aβ–CU group comparisons; age and global Tau‐MaX were used for AD+sLATE– versus AD+sLATE+ group comparisons. For longitudinal analyses, we fit a LME model at every voxel with random intercepts using the lmerNIfTI package in R^44^ and used the same equations as above, except global Tau‐MaX was used instead of temporal Tau‐MaX. For both cross‐sectional and longitudinal analyses, comparisons between CI and Aβ–CU groups are shown at *p* < 0.01 FWER‐ or FDR‐corrected to highlight only the most robust effects, as a *p* < 0.05 threshold yielded widespread significance. Comparisons between patient groups are shown at *p* < 0.05 FWER‐ or FDR‐corrected to retain sensitivity to more subtle group differences.

#### 2.4.4 Cognitive domain analyses

We evaluated group differences across three cognitive domains: memory, executive function, and language – using ADSP‐PHC composite scores provided by ADNI. Pairwise *t* tests were performed to assess group differences and ANOVA was performed when controlling for global Tau‐MaX in the models. For cross‐sectional analysis, we used the psychometric assessment closest to the MRI scan used in the imaging cross‐sectional analyses discussed earlier. For longitudinal analyses, we included all available assessments ±5 years from the time point used in the cross‐sectional analyses. LME models with random slopes and random intercepts were used. To assess differences in rates of decline between groups, we performed pairwise *t* tests on the interaction between time × group from the LME models. Covariates included are in the following model equation:

$$\begin{array}{ccc} & & \text{Domain}\;\text{score}∼\text{time}+\text{group}+\text{age}\;\text{at}\;\text{baseline}\;\text{assessment}\\ & & \;+\;\text{years}\;\text{of}\;\text{education}+\text{global}\;\text{Tau}-\text{MaX}\\ & & \;+\;\text{interval}\;\text{between}\;\text{baseline}\;\text{assessment}\;\text{and}\;\text{tau}-\text{PET}+\text{time}\\ & & \;\times \;\text{group}+(1+\text{time}|\text{ID}) \end{array}$$

#### 2.4.5 SILA analysis of cognitive trajectories

We applied the sampled iterative local approximation (SILA) algorithm^45^ to estimate the distribution of the age at which individuals become CI relative to the age of tau onset. To estimate age of tau onset, we applied the SILA model using global Tau‐MaX values from our previously described dataset^39^ following the approach outlined by Betthauser et al.^45^ Briefly, SILA estimates a biomarker trajectory by first using discrete sampling to quantify the rate of change at each biomarker level, followed by robust LOESS smoothing and numerical integration via Euler's method. Once a positivity threshold is defined, the resulting trajectory can be aligned to the time of biomarker positivity, allowing estimation of time from positivity for any given biomarker value. Tau positivity was defined using the 97.5th percentile of global Tau‐MaX values in Aβ–CU individuals, yielding a cut‐off of >3.31, and tau onset age was estimated based on the final event point with an extrapolation of 3 years and truncation of estimated ages to the ages observed in the original model. Then, for each patient group, we fit separate SILA models to cognitive measures to evaluate differences in cognitive trajectories. For these models, we anchored cognitive models to tau age (rather than chronological age) using the estimated age of tau onset. We then estimated that age of cognitive impairment onset using the group‐specific model final event point and extrapolation of 5 years and truncation of estimated ages to the ages observed in the original model. The threshold for cognitive impairment for each domain was defined using a threshold of 1.5 standard deviations (SD) below the mean of CU participants and scaled by –1 as SILA associates higher values with higher disease severity.

#### 2.4.6 Validation in autopsy‐confirmed cohort

Finally, we evaluated the quartile‐based enrichment strategy in an autopsy‐confirmed sample from ADNI. The HV cut‐off derived from Aβ+ patients in the main cohort were applied to categorize patients in the first quartile (severe hippocampal atrophy) and the third/fourth quartile (less severe hippocampal atrophy). Within each quartile‐based group, we assessed the frequency of ADNC with and without LATE‐NC from the neuropathologic assessments available in ADNI. Pointwise MTL thickness differences across AD+LATE– and AD+LATE+ groups were also assessed using GLM, controlling for age at scan, Braak stage, *ante mortem* interval (AMI), and field strength. Enrichment for LATE co‐pathology in the lowest quartile was evaluated using Fisher's exact test.

## 3 RESULTS

Applying a quartile‐based cut‐off on adjusted HVs in CI individuals from ADNI yielded 115 individuals with AD+sLATE– (Aβ+, Q3–Q4, volume > 2861.4 mm^3^), 58 with AD+sLATE+ (Aβ+, Q1, volume < 2502.2 mm^3^), and 20 with AD−sLATE+ (Aβ–, Q1). Summary characteristics are reported in Table 1. As expected by definition of the groups, AD+sLATE+ had significantly lower HVs compared to AD+sLATE– but did not differ from AD−sLATE+. AD+sLATE– and AD−sLATE+ had lower tau burden and load, as measured by temporal and global Tau‐MaX, compared to AD+sLATE+, suggesting some degree of greater AD progression in the AD+sLATE+ group than in the AD+sLATE– group.

**TABLE 1:** Demographics and cohort characteristics.

Mean (SD)	Aβ–CU (*n* = 193)	AD+sLATE− (*n* = 115)	AD−sLATE+ (*n* = 20)	AD+sLATE+ (*n* = 58)
Age	73.8 (7.1)	75.8 (8.0)	76.3 (7.7)	75.1 (8.0)
Sex (Male/Female)	110/93	65/50	15/5	33/25
Education	16.3 (2.3)	16.1 (2.5)	17.4 (2.4)^*^	15.3 (2.5)
MMSE	29.0 (1.2)	26.9 (2.6)^***^	25.9 (2.7)^*^	23.3 (4.2)
CDR‐SB	0.1 (0.3)	1.8 (1.7)^***^	3.3 (2.2)	4.2 (3.1)
Adjusted hippocampal volume	3282 (361)	3194 (241)^***^	2075 (292)^*^	2237 (229)
Centiloids	1.2 (9.2)	80.1 (37.1)^*^	−1.6 (11.9)^***^	97.7 (38.7)
MTL Tau‐MaX	0.5 (2.0)	21.4 (26.0)^***^	5.5 (7.8)^***^	44.2 (30.9)
Global Tau‐MaX	0.4 (1.7)	12.7 (18.6)^***^	2.5 (3.9)^***^	30.4 (28.2)

*Note*: Pairwise group comparisons are reported for AD+sLATE– versus AD+sLATE+ (asterisk in AD+sLATE– column) and AD−sLATE+ versus AD+sLATE+ group (asterisk in AD−sLATE+ column). Hippocampal volume is adjusted for age and intracranial volume.

Abbreviations: AD, Alzheimer's disease; CU, cognitively unimpaired; MMSE, Mini‐Mental State Examination; MTL, medial temporal lobe; Aβ, amyloid beta.

Significance levels are indicated as follows: ^*^ *p* ≤ 0.05; ^***^ *p* ≤ 0.001.

### 3.1 Region‐level analysis and imaging features

**FIGURE 1:**
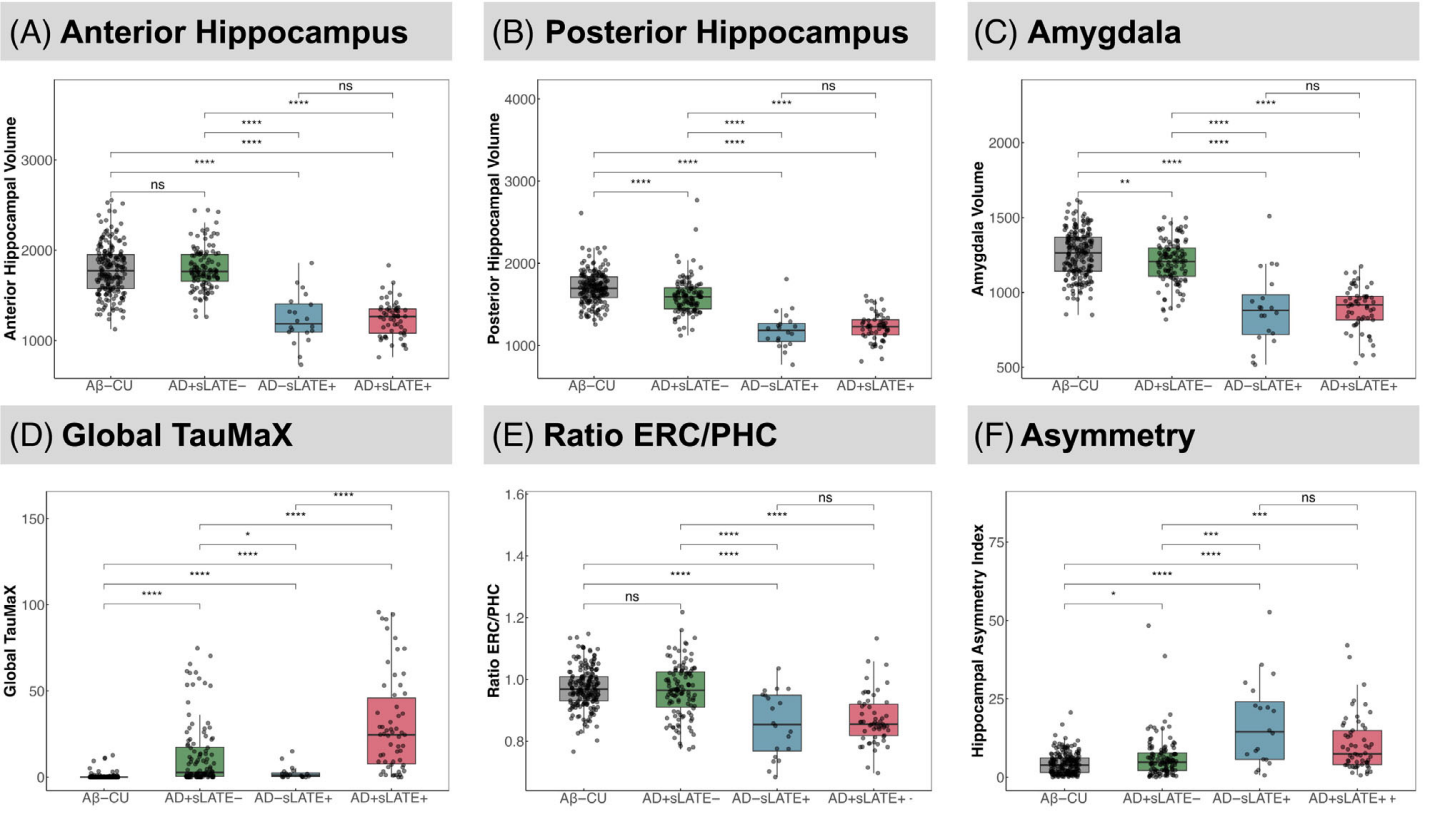
Differences between regions of interest (ROIs) and imaging features across suspected groups. (A–D) Distribution of anterior/posterior hippocampal volume, amygdala volume, and tau burden as measured by global Tau‐MaX from tau‐PET across groups. (E and F) Distribution of additional features suggestive of limbic‐predominant age‐related TDP‐43 encephalopathy (LATE), from prior work, ratio of entorhinal cortex (ERC) to parahippocampal cortex (PHC), and hippocampal asymmetry calculated as (|[L–R]|/[L+R]) × 200. Significance levels are indicated as follows: *p ≤ 0.05, **p ≤ 0.01, ***p ≤ 0.001, ****p ≤ 0.0001. ns, not significant (*p* > 0.05).

Plots in Figure 1A–C show that AD+sLATE– had lower mean PH volume, but not AH volume compared to Aβ–CU, while the AD+sLATE+ showed lower AH and PH as well as amygdala volumes, possibly indicating greater anterior involvement of TDP‐43, although AD+sLATE– displayed a significant amygdala difference, albeit significantly less so than the sLATE+ groups. Despite AD+sLATE– and AD+sLATE+ differing in tau load, AD+sLATE– covered a wide range of global Tau‐MaX with significant overlap with the AD+sLATE+ group (Figure 1D).

Our previous work showed that lower ERC/PHC ratio could discriminate TDP‐43‐positive individuals with intermediate/high ADNC from those without TDP‐43 with an area under the curve (AUC) of 0.82.^21^ Here, we found that AD+sLATE+ showed lower ERC/PHC ratio, similar to AD−sLATE+ (Figure 1E). Studies have shown that individuals with LATE also tend to show asymmetric atrophy,^10^ and we found that both AD+sLATE+ and AD−sLATE+ groups had greater asymmetry indices compared to AD+sLATE– and Aβ–CU groups (Figure 1F).

### 3.2 Pointwise analysis of MTL thickness

#### 3.2.1 Cross‐sectional

**FIGURE 2:**
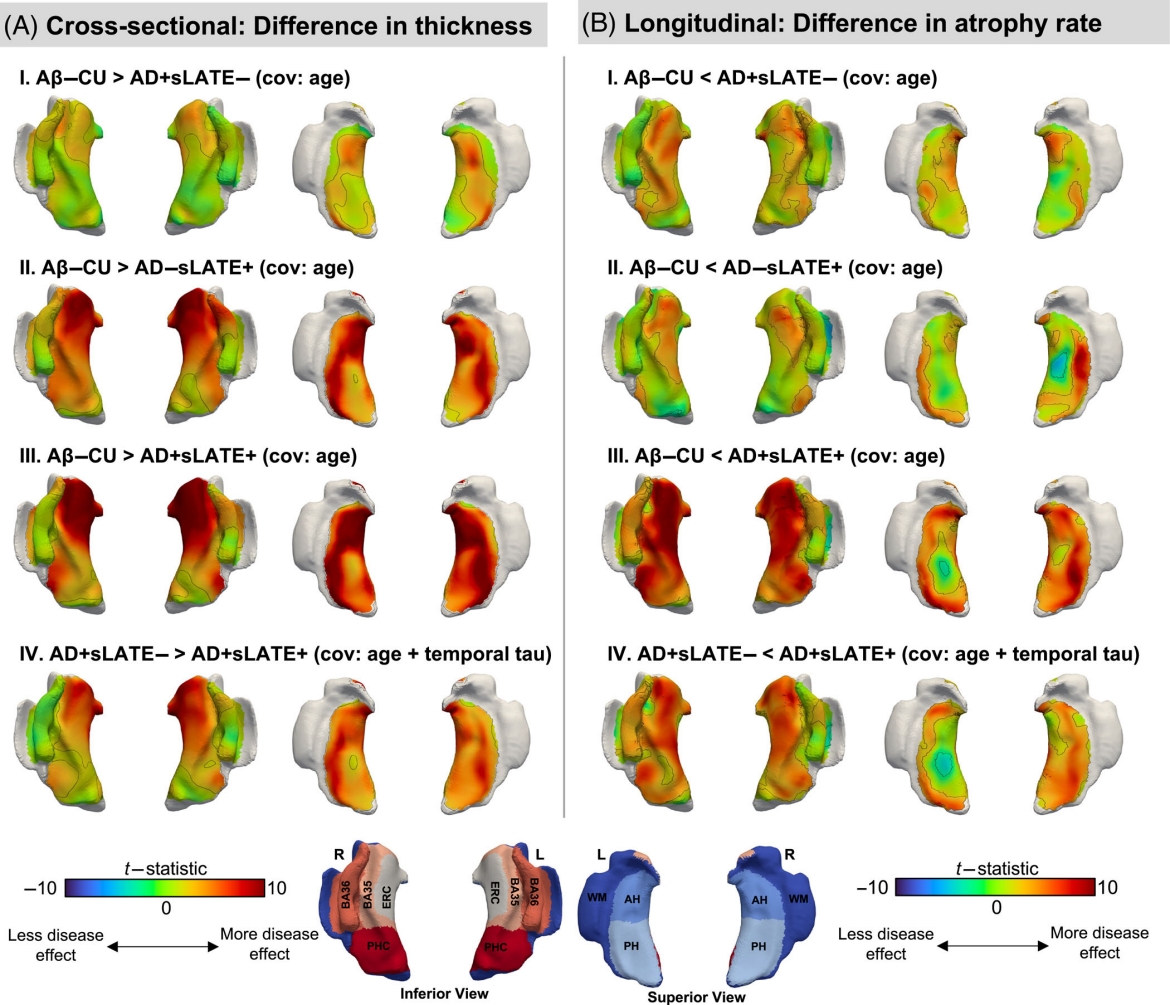
Pointwise structural cross‐sectional and longitudinal group differences in medial temporal lobe (MTL). (A) Maps represent group differences in thickness obtained using a surface‐based MTL analysis pipeline, CRASHS. Each 3D model represents the inflated representation of the mid‐surface between the MTL gray matter and white matter, shown either from superior or inferior. Age (I–IV) and temporal Tau‐MaX (IV) were used as covariates. Tau‐MaX is a measure that accounts for both the amount and extent of tau burden. Black outlines represent clusters significant at *p* < 0.05 family‐wise error rate‐corrected (using the threshold‐free cluster enhancement method with 10,000 permutations). (B) Linear mixed‐effects models were run at every point in the MTL thickness maps. For comparisons with cognitively unimpaired (I–III), the following equation was used: Thickness ∼ time + group + age at baseline scan + time × group + (1|ID). For comparisons between AD groups (IV), the following equation was used: Thickness ∼ time + group + age at baseline scan + temporal Tau‐MaX + interval between baseline MRI and tau PET + time × group + (1|ID). Black outlines represent clusters significant at false discovery rate‐corrected *p* < 0.05. AH, anterior hippocampus; BA35/36, Brodmann areas 35/36; CRASHS, cortical reconstruction for automatic segmentation of hippocampal subfields; ERC, entorhinal cortex; MRI, magnetic resonance imaging; PH, posterior hippocampus; PHC, parahippocampal cortex; WM, white matter.

Compared to Aβ–CU, AD+sLATE– showed lower thickness in the anterior ERC and BA35 when controlling for age; however, AD−sLATE+ and AD+sLATE+ showed stronger effects in these regions with a clear anterior‐to‐posterior gradient in the MTL cortex (Figure 2A I–III). Controlling for AD progression with temporal Tau‐MaX, the AD+sLATE+ group displayed lower thickness in the anterior regions of the MTL compared to the AD+sLATE– group (Figure 2A IV).

#### 3.2.2 Longitudinal

Compared to Aβ–CU, AD+sLATE– showed limited atrophy throughout the MTL cortex, while AD−sLATE+ showed atrophy confined to the ERC and BA35; AD+sLATE+ showed severe atrophy throughout the MTL cortex, as well as the hippocampus (Figure 2B I–III). Additionally, when comparing AD+sLATE– to AD+sLATE+, controlling for temporal Tau‐MaX at baseline, AD+sLATE+ displayed a significantly greater rate of atrophy, particularly within cortical MTL regions (Figure 2B IV).

### 3.3 Voxel‐wise analysis of whole‐brain thickness

#### 3.3.1 Cross‐sectional

**FIGURE 3:**
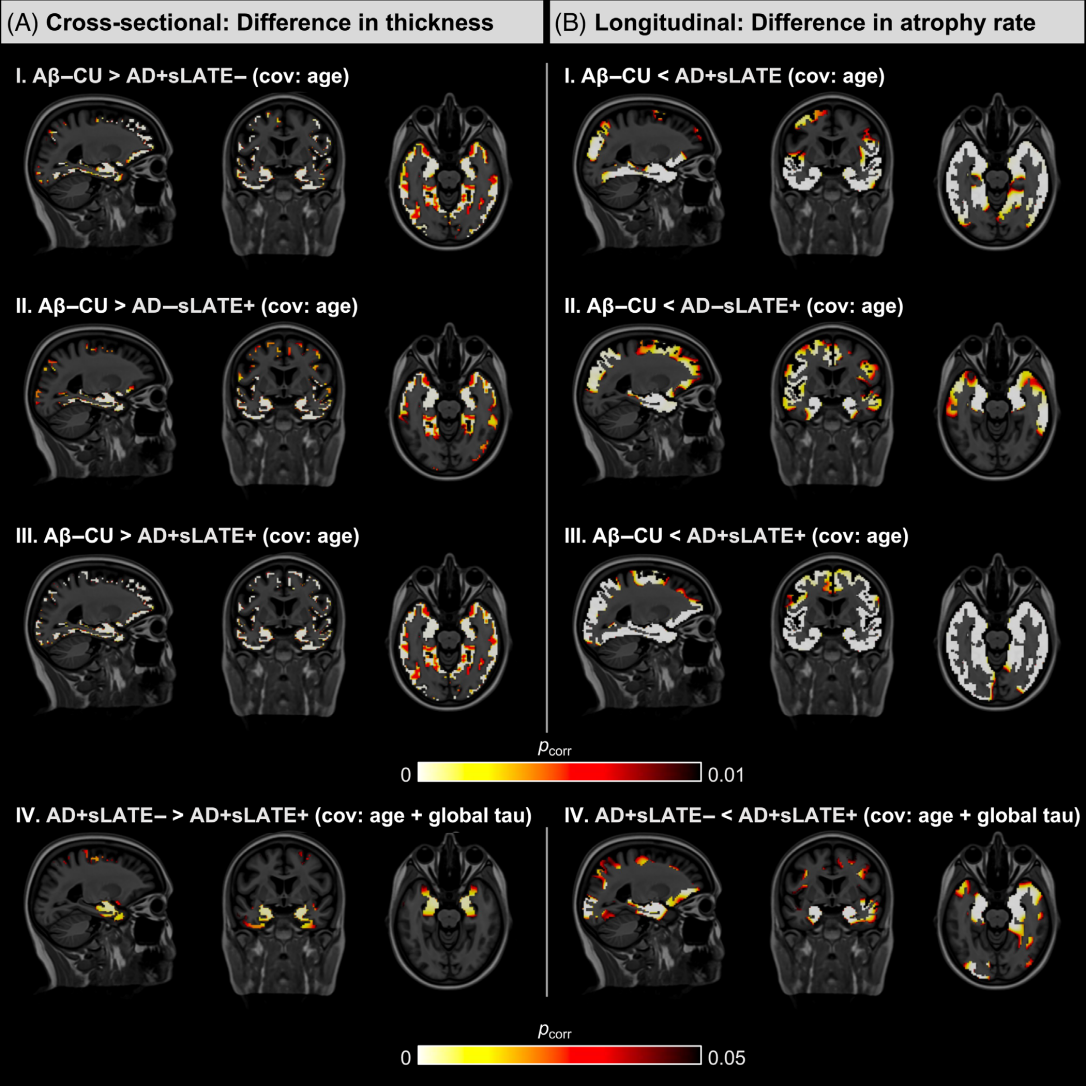
Voxel‐wise structural cross‐sectional and longitudinal group differences in whole brain. (A) Voxel‐wise group comparisons in whole‐brain thickness maps for controls versus patient groups (I–III) are shown at *p* < 0.01 family‐wise error rate (FWER)‐corrected and between patient groups (IV) are shown at *p* < 0.05 FWER‐corrected. Age (I–IV) and global Tau‐MaX (IV) at baseline were used as covariates. Tau‐MaX is a measure that accounts for both the amount and extent of tau burden. (B) Linear mixed‐effects models were run at every voxel in the whole‐brain thickness maps. For comparisons with cognitively unimpaired (CU) (I–III), the following equation was used: Thickness ∼ time + group + age at baseline scan + time × group + (1|ID). For comparisons between Alzheimer's disease (AD) groups (IV), the following equation was used: Thickness ∼ time + group + age at baseline scan + global Tau‐MaX + interval between baseline MRI and tau PET + time × group + (1|ID). The *p* values of the interaction between group × time for comparisons with CU (I–III) are shown at *p* < 0.01 false discovery rate (FDR)‐corrected, and between AD groups (IV) are shown at *p* < 0.05 FDR‐corrected.

Compared to Aβ–CU, AD+sLATE– showed lower thickness in the temporal and parietal regions, consistent with typical AD. AD−sLATE+ showed lower thickness largely confined to the medial and anterior temporal and fronto‐insular regions. AD+sLATE+ showed lower thickness throughout the cortex (Figure 3A I–III). Interestingly, when comparing AD+sLATE– and AD+sLATE+ and controlling for global Tau‐MaX to account for AD severity, AD+sLATE+ displayed lower thickness almost exclusively in the medial and anterior temporal lobe regions at *p* < 0.05 FDR‐corrected threshold similar to that of AD−sLATE+ (Figure 3A IV).

#### 3.3.2 Longitudinal

Results were generally consistent with cross‐sectional findings, though they revealed a more extensive pattern of significant effects. When compared to Aβ–CU, AD+sLATE– displayed a significantly higher rate of atrophy in the temporal and parietal regions, AD−sLATE+ in the temporal lobe, and AD+sLATE+ throughout the cortex (Figure 3B I–III). Comparison between AD+sLATE– and AD+sLATE+ when controlling for Tau‐MaX and for the interval between tau‐PET and the baseline MRI scan in addition to age again revealed that the AD+sLATE+ group had a greater rate of atrophy than AD alone, most prominently and largely confined to medial and anterior temporal lobe regions, as well as orbitofrontal (Figure 3B IV). Nonetheless, there was some evidence of posterior cortical involvement as well.

### 3.4 Differences between groups across cognitive domains

#### 3.4.1 Cross‐sectional

**FIGURE 4:**
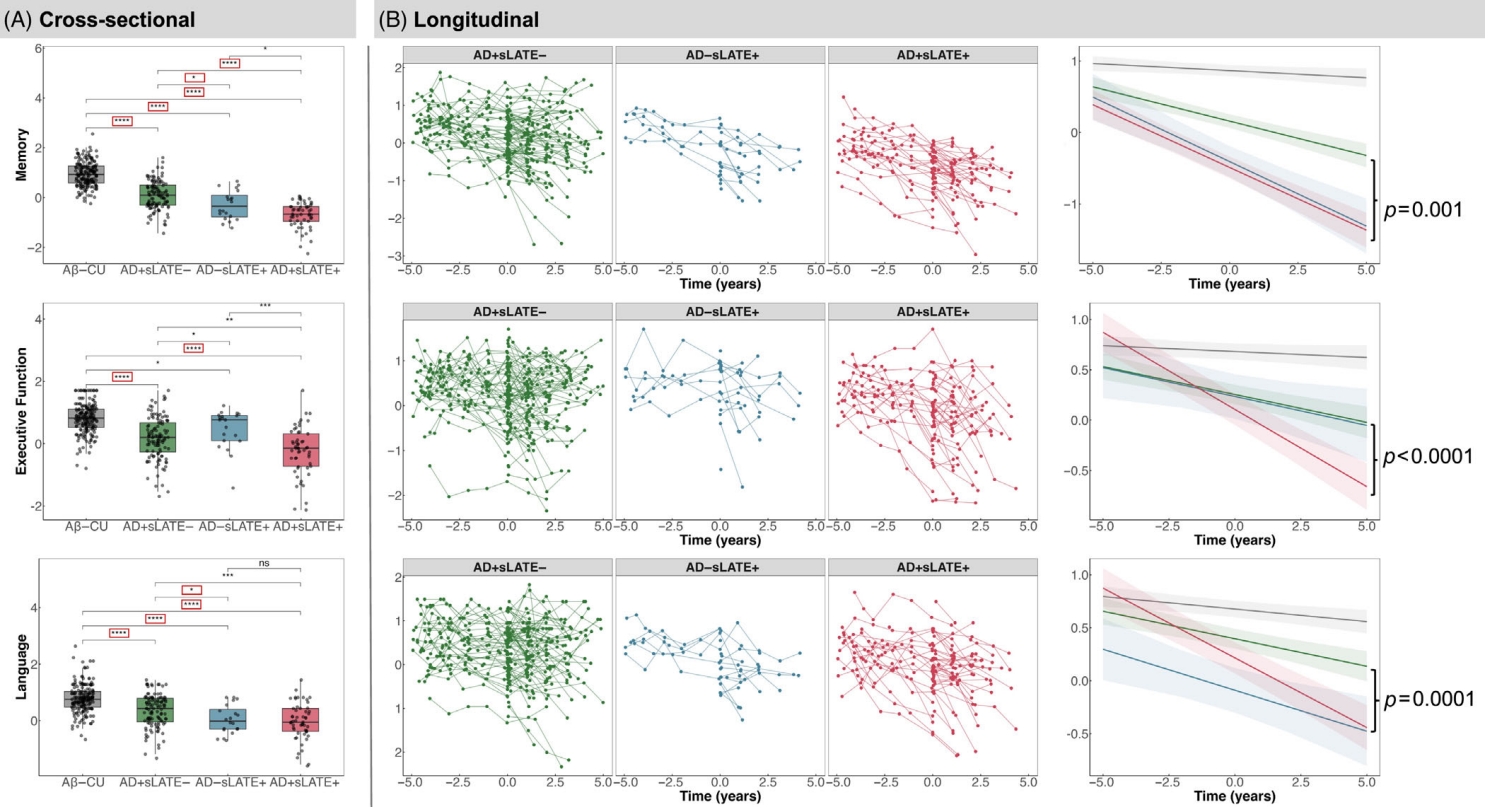
Cross‐sectional and longitudinal differences in cognitive domains and trajectories. (A) Pairwise group differences (*p < 0.05, ***p < 0.001, ****p < 0.0001) at baseline. Asterisks outlined in red reflect significant differences when controlling for global Tau‐MaX. Notably, baseline differences in language and executive function between AD−sLATE+ and AD+sLATE+ groups were attenuated and no longer significant after controlling for tau burden. (B) Linear mixed‐effects model controlling for global Tau‐MaX and time difference in years across three cognitive domains. Domain score ∼ time + group + age at baseline assessment + years of education + global Tau‐MaX + interval between baseline assessment and tau PET + time × group + (1 + time|ID). Model allows for random slopes and intercepts. Year 0 represents the “cross‐sectional” time point that is anchored to amyloid‐ and tau‐PET imaging. *p*‐values on the rightmost plot represent significant differences tested between the slopes of AD+sLATE− and AD+sLATE+.

Groups differed from each other in composite memory score. AD+sLATE+ had the lowest score, followed by AD−sLATE+, AD+sLATE–, and then Aβ–CU. Furthermore, when controlling for global tau, AD+sLATE+ differed from AD+sLATE– and AD–sLATE+, suggesting memory impairment beyond the effect of tau‐related neurodegeneration. Pairwise comparisons revealed significant differences in executive function between all groups. However, AD+sLATE+ no longer significantly differed from AD+sLATE– or AD−sLATE+ when controlling for global tau, likely indicating minimal effect of non‐tau related neurodegeneration on executive function. Finally, all groups, except AD–sLATE+ versus AD+sLATE+, also showed significant differences in language. However, when controlling for tau, the difference between AD+sLATE– versus AD+sLATE+ was no longer significant (Figure 4A). Thus, this suggests that once the effects of AD severity based on tau are accounted for, the only remaining cross‐sectional difference is in memory, which is primarily affected by LATE.

#### 3.4.2 Longitudinal

Longitudinal trajectories across the three cognitive domains were assessed using LME models, adjusting for global tau burden. In the memory domain, AD+sLATE+ had a decline similar to that of AD−sLATE+, but steeper than AD+sLATE–. For executive function, AD+sLATE– and AD−sLATE+ followed a similar trajectory, but AD+sLATE+ showed faster decline. Interestingly, in the language domain, the AD−sLATE+ group started out with lower composite scores than both AD+sLATE+ and AD+sLATE–, but while AD+sLATE+ declined more rapidly over time, AD−sLATE+ showed a slower progression, with rates of decline more comparable to the AD+sLATE– group (Figure 4B).

**FIGURE 5:**
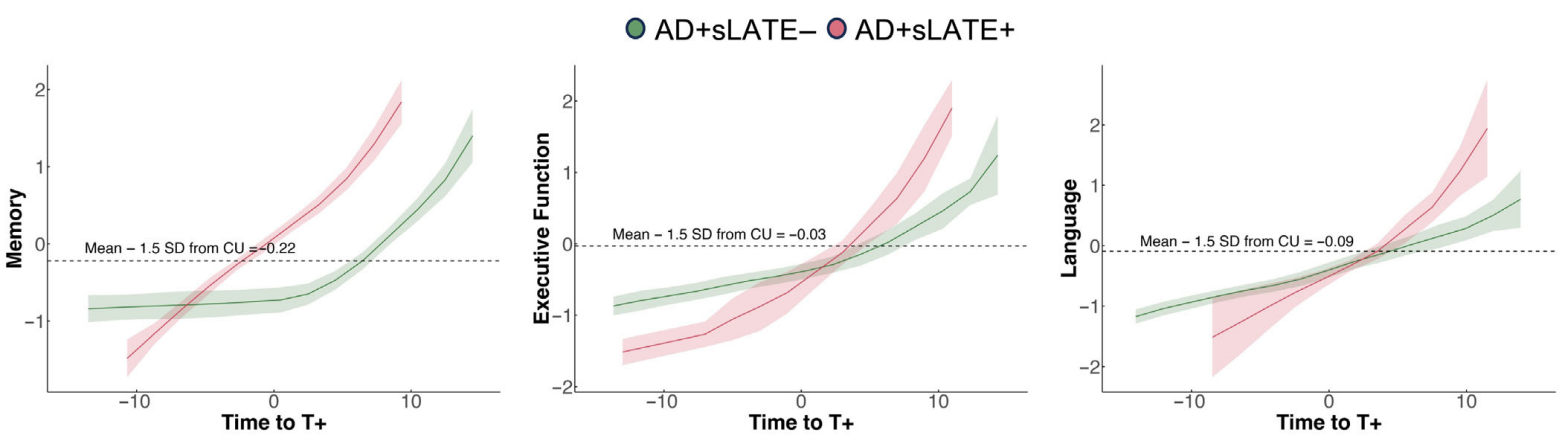
Estimated time to domain‐specific cognitive impairment using sampled iterative local approximation (SILA) modeling. Time to tau positivity (T+) was first estimated using SILA and then anchored to the age at neuropsychological exam. (A) Separate SILA curve was then fit for each group and cognitive domain. The impairment threshold (dashed line) was defined as the mean minus 1.5 standard deviations from cognitively unimpaired (CU) individuals in the Alzheimer's Disease Neuroimaging Initiative (ADNI). Composite scores were scaled by −1 so that higher values reflect greater impairment, consistent with SILA's assumptions. Cognitive trajectories anchored to tau onset age should be interpreted only for groups with tau positivity, that is, AD+sLATE− and AD+sLATE+.

SILA analyses examining cognitive decline across the three domains, anchored to estimated age of tau onset (Section 2.4.5), are presented in Figure 5 to illustrate group differences in cognitive trajectories between AD+sLATE– and AD+sLATE+ individuals. All composite scores were scaled by –1 in the SILA models, as SILA associates higher values with greater disease severity; thus, more positive scores indicate greater impairment. Thresholds for cognitive impairment were defined as 1.5 SD above the mean of CU: –0.22 for memory, –0.03 for executive function, and –0.09 for language. When aligning groups along the AD continuum based on estimated age of tau positivity (T+), distinct cognitive trajectories emerged across domains. In the memory domain, individuals with AD+sLATE+ exhibited earlier and more rapid decline than those with AD+sLATE–, with divergence beginning years before tau positivity – suggesting contributions from non‐AD pathology first. In contrast, executive function trajectories were relatively similar between groups prior to T+, but the AD+sLATE+ group demonstrated a clear inflection point at tau onset, followed by accelerated decline compared to the AD+sLATE– group. Language performance showed a pattern similar to executive function: While both groups followed a comparable course before T+, the AD+sLATE+ group experienced a steeper decline after crossing the tau positivity threshold. Together, these findings highlight that the presence of LATE co‐pathology may drive earlier, pre‐tau, memory impairment, and more aggressive cognitive deterioration across multiple domains after tau burden becomes substantial.

In addition to aligning trajectories to estimated tau onset (T+), we also examined cognitive trajectories using chronological age at the time of neuropsychological assessment, irrespective of tau status. This approach allowed us to assess domain‐specific trajectories across groups without assuming a common pathological anchor point, which is particularly important given that the AD−sLATE+ group does not exhibit tau pathology and therefore lacks a meaningful T+ reference. Notably, under this framework, the AD−sLATE+ group showed more rapid decline similar to AD+sLATE+ in both memory and language domains, further highlighting the LATE‐like cognitive profile of the AD+sLATE+ group (Figure S2).

### 3.5 Enrichment in autopsy‐proven cohort

**FIGURE 6:**
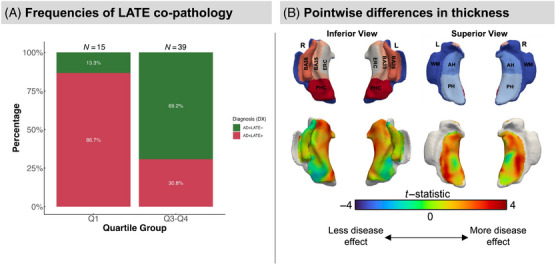
Validation in autopsy cohort using baseline structural magnetic resonance imaging (MRI). (A) Frequencies of limbic‐predominant age‐related transactive response DNA‐binding protein 43 (TDP‐43) encephalopathy (LATE) co‐pathology occurrence in low‐hippocampal‐volume (HV) groups in autopsy‐confirmed cases. Q1 represents the group with significant hippocampal atrophy and Q3–Q4 represents the group with less severe hippocampal atrophy. (B) Structural thickness differences in baseline MRI comparing intermediate/high AD neuropathological change (ADNC) with and without LATE neuropathic change (LATE‐NC) stage 2 (hippocampal TDP‐43), that is, AD+LATE− > AD+LATE+. Covariates included are age, Braak stage, *ante mortem* interval, and field strength (1.5T/3T).

Among the 79 ADNI participants with autopsy‐confirmed neuropathological diagnosis of intermediate/high ADNC, 15 were assigned to the Q1 quartile group and 39 to the Q3–Q4 quartile group based on their adjusted HV at baseline MRI. The AMI, that is, time between baseline MRI and death, for these 54 participants was 7.2 ± 3.5. Within Q1, 13 of the 15 (86.7%) individuals had autopsy‐confirmed diagnosis of LATE‐NC (stage 1, 2, or 3), whereas in Q3–Q4, 13 of the 39 (30.8%) individuals had a LATE‐NC diagnosis (Figure 6A). AD+LATE+ was significantly enriched in the lowest quartile compared to Q3–Q4 (Fisher's exact test, OR = 13.8, *p* < 0.001). These findings underscore that severe hippocampal atrophy seen beyond “typical” AD is associated with concomitant LATE, particularly early in symptomatic disease. Pointwise MTL analyses on baseline MRI revealed expected spatial patterns with autopsy‐confirmed AD+LATE+ showing lower thickness in the anterior MTL, particularly the ERC and BA35, compared to autopsy‐confirmed AD+LATE– when controlling for Braak stage (Figure 6B). Lastly, the ERC/PHC ratio in the autopsy‐confirmed AD+LATE– and AD+LATE+ groups also differed, with AD+LATE+ demonstrating lower ratios, suggesting more anterior atrophy and consistent with our findings in the clinically defined groups (Table S1).

Informed by prior *post mortem* imaging work showing that AD+LATE+ cases are enriched among individuals with the smallest HVs,^25^ our goal was to maximize specificity by focusing on the bottom quartile as a proof‐of‐concept design to identify individuals most likely to harbor concomitant LATE pathology. Exploratory ROC analyses within the autopsy cohort (AUC = 0.74; Figure S3) yielded an optimal threshold based on the Youden index near the 47th percentile of age‐ and ICV‐adjusted HV, achieving balanced specificity (0.71) and sensitivity (0.76). Although this threshold improved sensitivity, it reduced specificity relative to the quartile‐based cut‐off (25th percentile: specificity = 0.95, sensitivity = 0.32). This trade‐off highlights that higher thresholds capture more true positives but increase false positives. Given our objective to define a group with high confidence for LATE co‐pathology, the quartile‐based approach – while conservative – was preferred.

## 4 DISCUSSION

This study demonstrates that a simple, quartile‐based cut‐off of HV effectively identifies a subgroup of CI individuals on the AD continuum likely enriched for concomitant LATE and associated with a more aggressive disease course. This stratification approach uses a single MRI‐derived metric to group individuals in a way that meaningfully predicts both brain atrophy patterns and cognitive prognosis. The simplicity adds to ease of implementation in both research settings and routine clinical care. The motivation for specifically using HV as a probabilistic marker for concomitant LATE emerges from *post mortem* imaging work in which HV is significantly reduced in the setting of LATE with or without concomitant AD.^25^

While other metrics such as the ERC/PHC ratio^21^ and tau‐to‐neurodegeneration (T/N) mismatch^27^, ^28^ have been proposed as markers of suspected LATE, we chose to focus on whole HV for both biological and practical reasons. HV is generally considered a more robust structural measure, particularly in large‐scale or heterogeneous datasets. Additionally, T/N mismatch requires tau‐PET imaging, which may not be available in many clinical or trial settings, and PET‐based approaches like FDG‐PET^22^, ^23^, ^24^ face similar constraints. In contrast, HV can be derived from a single structural MRI scan, increasing its potential for integration into both research pipelines and clinical workflows.

Using this cut‐off, we found that individuals in the lowest HV quartile (Q1) displayed significantly different imaging and cognitive profiles from those in the higher quartiles. The AD+sLATE+ group – defined by low HV within biomarker‐confirmed AD – showed pronounced anterior MTL thinning and reduced amygdala volume, consistent with prior reports identifying these regions as early TDP‐43 sites.^11^, ^46^, ^47^ The ERC/PHC ratio, a metric previously demonstrated to discriminate between cases of AD with versus without TDP‐43 in a *post mortem* series,^21^ was also lower in the AD+sLATE+ group. Notably, the AD−sLATE+ group had a similarly reduced ratio compared to the AD+sLATE– group. In addition, both AD+sLATE+ and AD−sLATE+ groups exhibited higher asymmetry indices, supporting literature linking LATE with asymmetric atrophy patterns.

Beyond the MTL, the AD+sLATE+ group displayed more widespread cortical thinning, particularly in regions associated with LATE, such as medial‐anterior temporal polar and orbitofrontal cortex and anterior extrahippocampal region, paralleling patterns reported across *ex vivo* MRI,^48^ in vivo MRI, and FDG‐PET studies of LATE‐NC.^23^, ^49^, ^50^ This consistency across imaging modalities and pathological studies reinforces the biological plausibility of the phenotype observed here as reflecting suspected TDP‐43 co‐pathology. Longitudinally, AD+sLATE+ exhibited faster cortical thinning than either AD+sLATE– or AD−sLATE+, particularly in anterior MTL and temporal polar regions, even after adjusting for tau burden – reinforcing the likely role of co‐pathology rather than AD alone in driving this difference.

The cognitive differences between these groups largely mirrored expectation based on the imaging. AD+sLATE+ individuals had lower memory and language performance at baseline and declined more rapidly over time. Remarkably, memory decline in this group appeared to precede estimated tau positivity. As cognitive decline generally does not appear prior to tau burden measurable with PET, this result suggests that LATE, or another non‐AD pathology, is likely to contribute to these memory changes prior to manifest clinical AD in at least a subset of these patients. For language and executive domains, the AD+sLATE+ group followed a similar trajectory to AD+sLATE– prior to tau positivity but had an accelerated drop‐off after reaching tau thresholds – indicating a potential synergy between AD and TDP‐43 pathologies in driving decline. This finding is also consistent with LATE having its earliest and most prominent effect on memory.^15^

According to biomarker models of the AD continuum,^51^ cognitive decline typically emerges only after tau burden becomes measurable with PET. This framework is further supported by longitudinal work showing that progression from preclinical AD to MCI and dementia occurs almost exclusively in individuals with evidence of at least medial temporal and more frequently inferolateral temporal tau deposition on PET imaging.^52^ Therefore, impairment at the A+T– stage is unlikely to be driven by typical AD progression and may instead reflect non‐AD or mixed pathology such as LATE. Consistent with this idea, recent studies have reported an increasing prevalence of A+T– MCI with advancing age,^53^, ^54^ perhaps due to the fact that LATE or other non‐AD pathologies, which are more common with advanced age, drive cognitive impairment in the absence of detectable tau pathology. It is worth noting that the current results are largely inconsistent with the notion that the lowest quartile can be explained simply by AD severity marked by tau burden based on at least three findings: (1) cognitive and structural imaging differences persisted after controlling for tau, (2) additional analyses estimating time to tau positivity – an approach that aligns individuals by biological stage – showed domain‐specific differences even prior to tau onset, and (3) autopsy validation confirmed enrichment for TDP‐43 pathology.

Interestingly, within our ADNI sample, we did not observe significant age differences between AD+sLATE+ and AD+sLATE– groups. While it is unclear why this may be the case, it may reflect ADNI's relatively younger participants compared to community autopsy studies. Further, as our analyses were anchored within the AD continuum, the effect of age may have been attenuated by the fact that AD severity is also a key risk for LATE co‐pathology.

Importantly, we further validated the quartile‐based HV threshold in an independent autopsy‐confirmed cohort drawn from earlier ADNI phases that did not include tau‐PET imaging. This analysis served as neuropathologic validation of the in vivo stratification. Nearly 90% of individuals in the lowest HV quartile had confirmed AD+LATE+, whereas only about 10% of ADNC without LATE‐NC cases met this threshold. As symptomatic patients in ADNI are enrolled at either the MCI or mild dementia stage, and some were even CU in this autopsy group, this supports the notion that severe atrophy at early disease stages is enriched in those with concomitant LATE. As this population overlaps with those eligible for US Food and Drug Administration‐approved anti‐amyloid therapies, the threshold developed here may be a useful tool for stratifying patients and determining whether suspected LATE co‐pathology influences treatment outcomes. Such stratification could begin to clarify whether concomitant LATE modulates the benefits or risks of disease‐modifying therapies, which could then influence decisions about treatment.

While the quartile‐based HV cut‐off used here clearly enriches in the proportion of individuals with concomitant LATE, it does not definitively distinguish the presence of TDP‐43 co‐pathology from other potential drivers of hippocampal atrophy, for example, non‐AD pathologies, such as argyrophilic grain disease (AGD), frontotemporal lobar degeneration (FTLD), including other TDP‐43 pathologies, and cerebrovascular disease. Alternatively, the so‐called limbic variant of AD may also present with more severe MTL atrophy. Incorporation of the cut‐off described here with other clinical features from the recently proposed LATE criteria may result in further specificity. In particular, disproportionate hippocampal atrophy relative to MTL tau burden measured by tau‐PET may reduce the likelihood of limbic variant AD. These limitations underscore the ongoing need for validated in vivo biomarkers specific to TDP‐43 to improve pathological specificity.

This study has several limitations. While we validated our stratification approach using autopsy‐confirmed cases, the cohort size was modest, and the cut‐off was derived cross‐sectionally, which may not fully capture the longitudinal progression of LATE‐related neurodegeneration. Additionally, this work was conducted in ADNI – a highly selected research cohort – and findings may not generalize to more diverse or community‐based populations, where the prevalence and manifestation of co‐pathology may differ. Moreover, absolute HV thresholds are influenced by scanner vendor, field strength, acquisition protocol, and the segmentation software/pipeline used. While ADNI data are acquired using harmonized protocols, thresholds will need to be recalibrated for each dataset. Thus, our results should be interpreted as a proof‐of‐concept demonstration of a quartile‐based approach that enriches in the proportion of individuals with suspected LATE, rather than as a universally applicable cut‐off. The current threshold serves as a pragmatic framework to identify a high‐risk subgroup. Future studies with larger autopsy datasets could refine these cut points and potentially adopt two‐threshold strategies, as is common in other biomarker fields,^55^ with intermediate cases (Q2) representing an indeterminate zone requiring additional biomarkers or longitudinal follow‐up.

In the absence of molecular biomarkers for TDP‐43, structural imaging measures offer a probabilistic link to LATE, even in the presence of AD pathology. As indicators of downstream neurodegeneration, these measures provide not only a signal of potential co‐pathology but also insight into disease stage, based on the pattern and severity of atrophy. This added dimension may support clinical decision‐making, enhance patient stratification, and inform therapeutic targeting, particularly in trials where identifying individuals with mixed pathology is essential. The core contribution of this work lies in demonstrating that a simple, normative MRI‐based threshold – readily implementable across clinical and research settings – can yield biologically and clinically meaningful groupings. This approach offers a scalable strategy for enriching clinical trial cohorts, flagging high‐risk individuals in practice, and improving our understanding of the complex interplay between co‐pathologies in neurodegenerative disease. Depending on the therapeutic objective, this stratification method could be used to either selectively exclude individuals with suspected mixed pathology in trials seeking pure‐AD cohorts or intentionally include them when targeting the broader biological complexity of AD with co‐existing LATE.

## CONFLICT OF INTEREST STATEMENT

Nidhi S. Mundada, Xueying Lyu, Christopher A. Brown, Niyousha Sadeghpour, Emily McGrew, Philip A. Cook, James Gee, and Paul A. Yushkevich declare no competing interests. Long Xie is a paid employee of Siemens Healthineers. Sandhitsu R. Das has received consulting fees from Rancho Biosciences and NIA Therapeutics. David A. Wolk has served as a paid consultant for Eli Lilly and Beckman Coulter. He has also served on the DSMB for Functional Neuromodulation and GSK. He has received research support paid to his institution by Biogen. Author disclosures are available in the supporting information.

## CONSENT STATEMENT

We confirm that all human subjects provided informed consent. Human brain specimens were obtained in accordance with the State of Pennsylvania and University of Pennsylvania Institutional Review Board guidelines. Where possible, pre‐consent during life and, in all cases, next‐of‐kin consent at death were given.

## Supporting information



Supporting Information

ICMJE Disclosure Form
