# Supplementary material for: Enrichment of patients with concomitant limbic‐predominant age‐related TDP‐43 encephalopathy (LATE) on the Alzheimer's disease continuum using hippocampal volume
Source: Alzheimers Dement. 2025 Dec 13;21(12):e70970. doi: 10.1002/alz.70970 (PMC12701520; doi:10.1002/alz.70970)
Supplement: Supplementary file 1 — Supporting Information [file ALZ-21-e70970-s002.docx]

**Supplementary Figure 1: Cohort Flowchart.**


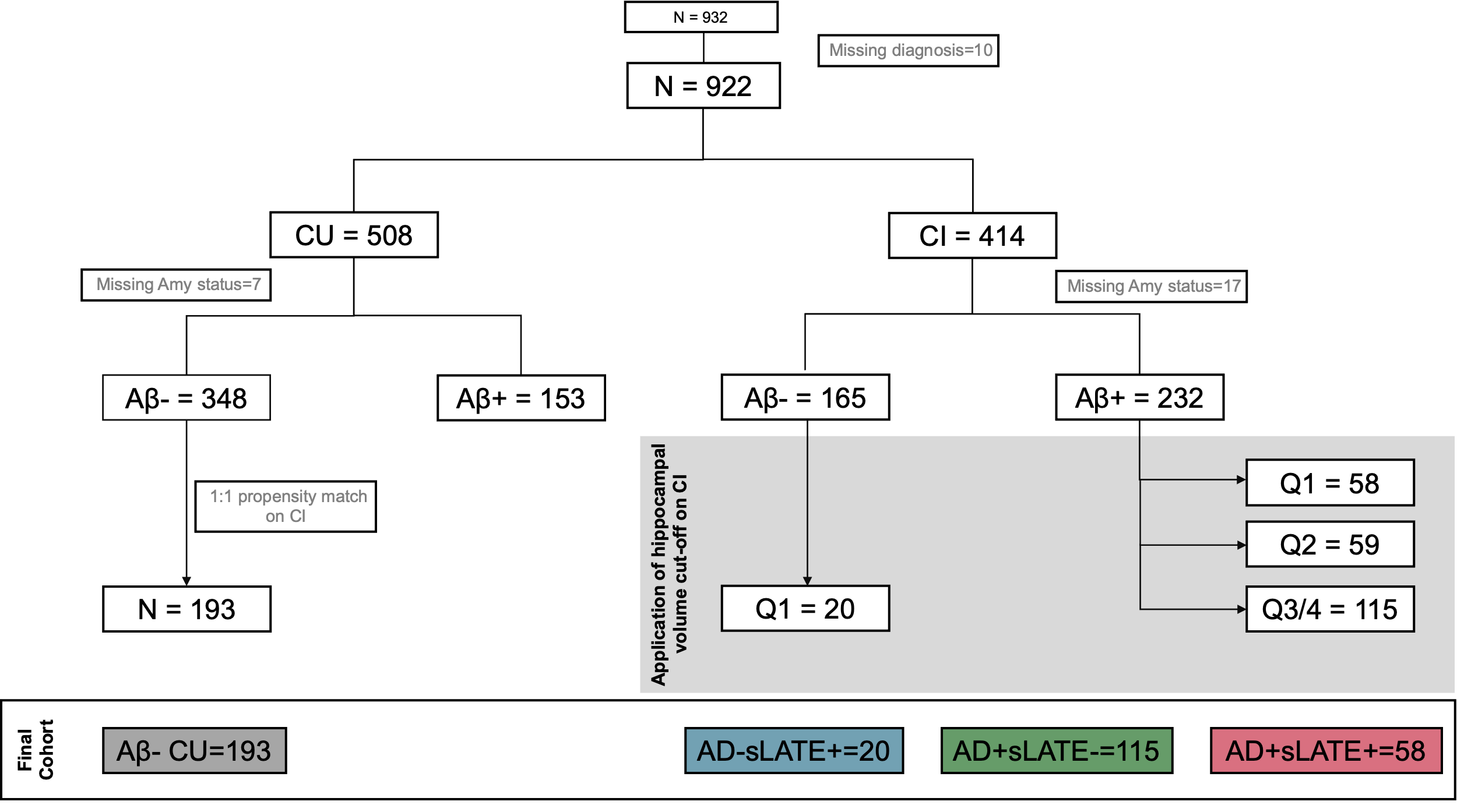


**Supplementary Figure 2: Estimated progression to domain-specific cognitive impairment across chronological age using SILA modeling.**


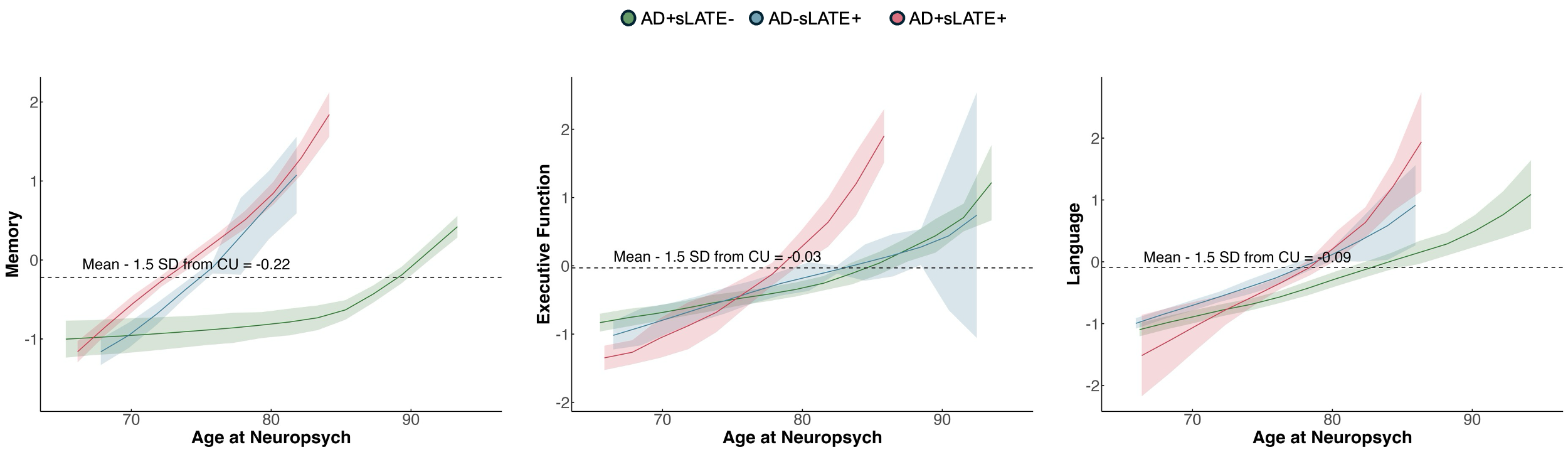


|  | **AD+LATE+** | **AD+LATE-** |
| --- | --- | --- |
| **Vol<25^th^ percentile** | 13 | 2 |
| **Vol>25^th^ percentile** | 12 | 27 |

**Supplementary Figure 3: ROC analysis in autopsy cases**


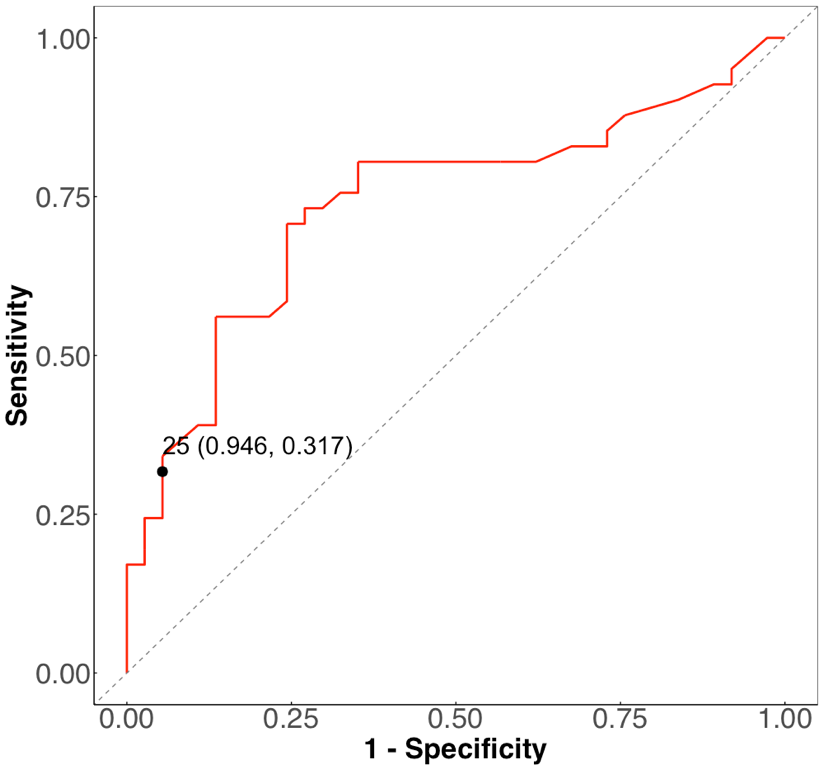


*Receiver operating characteristic (ROC) curve illustrating classification performance of age and ICV-adjusted hippocampal volume for distinguishing AD+LATE+ from AD+LATE– individuals in the ADNI autopsy cohort. The area under the curve (AUC = 0.74) indicates good discrimination. The filled circle marks the 25^th^-percentile (Q1) threshold used in the main analyses (specificity = 0.95, sensitivity = 0.32), selected to maximize specificity and define a well-characterized group enriched for LATE. The optimal Youden index threshold (~47^th^ percentile) achieved balanced performance (specificity = 0.71, sensitivity = 0.76) but was not used for the primary classification.*

**Supplementary Table 1: Demographics and Imaging Features in Autopsy Cohort.**

| mean (SD) | **AD+LATE-**  **(n=44)** | **AD+LATE+**  **(n=35)** | **p-value** |
| --- | --- | --- | --- |
| Age | 75.5 (7.5) | 78.0 (6.5) | ns |
| Sex (M/F) | 30/12 | 23/11 | ns |
| Education | 16.5 (2.6) | 15.9 (2.9) | ns |
| Adjusted Hippocampal Vol | 3031 (414) | 2707 (484) | 0.002 |
| Anterior Hippocampus Vol | 1629 (234) | 1495 (318) | 0.038 |
| Posterior Hippocampus Vol | 1480 (235) | 1391 (236) | ns |
| Amygdala Volume | 1097 (169) | 1057 (227) | ns |
| Braak Stage (3/4/5/6) | 3/3/25/11 | 3/4/21/6 | ns |
| Ratio ERC/PHC | 0.951 (0.1) | 0.898 (0.08) | 0.018 |
| Hippocampal Asymmetry Index | 7.3 (5.7) | 13.0 (13.3) | 0.014 |
